# Common Variants in *CLDN2* and *MORC4* Genes Confer Disease Susceptibility in Patients with Chronic Pancreatitis

**DOI:** 10.1371/journal.pone.0147345

**Published:** 2016-01-28

**Authors:** Anil K. Giri, Shallu Midha, Priyanka Banerjee, Ankita Agrawal, Syed Jafar Mehdi, Rajan Dhingra, Ismeet Kaur, Ramesh Kumar G., Ritika Lakhotia, Saurabh Ghosh, Kshaunish Das, Samir Mohindra, Surinder Rana, Deepak K. Bhasin, Pramod K. Garg, Dwaipayan Bharadwaj, INDIPAN and INDICO Consortium

**Affiliations:** 1 Genomics and Molecular Medicine Unit, CSIR-Institute of Genomics and Integrative Biology, New Delhi, India; 2 Academy of Scientific and Innovative Research, CSIR-Institute of Genomics and Integrative Biology Campus, New Delhi, India; 3 Department of Gastroenterology, All India Institute of Medical Sciences, New Delhi, India; 4 Human Genetics Unit, Indian Statistical Institute, Kolkata, India; 5 Department of Gastroenterology, Institute of Post Graduate Medical Education and Research, Kolkata, India; 6 Department of Gastroenterology, Sanjay Gandhi Post-graduate Institute of Medical Sciences, Lucknow, India; 7 Department of Gastroenterology, Post-graduate Institute of Medical Education & Research, Chandigarh, India; Odense University Hospital, DENMARK

## Abstract

A recent genome-wide association study (GWAS) identified association with variants in X-linked *CLDN2* and *MORC4*, and *PRSS1-PRSS2* loci with chronic pancreatitis (CP) in North American patients of European ancestry. We selected 9 variants from the reported GWAS and replicated the association with CP in Indian patients by genotyping 1807 unrelated Indians of Indo-European ethnicity, including 519 patients with CP and 1288 controls. The etiology of CP was idiopathic in 83.62% and alcoholic in 16.38% of 519 patients. Our study confirmed a significant association of 2 variants in *CLDN2* gene (rs4409525—OR 1.71, *P* = 1.38 x 10^-09^; rs12008279—OR 1.56, *P* = 1.53 x 10^-04^) and 2 variants in *MORC4* gene (rs12688220—OR 1.72, *P* = 9.20 x 10^-09^; rs6622126—OR 1.75, *P* = 4.04x10^-05^) in Indian patients with CP. We also found significant association at *PRSS1-PRSS2* locus (OR 0.60; *P* = 9.92 x 10^-06^) and *SAMD12-TNFRSF11B* (OR 0.49, 95% CI [0.31–0.78], P = 0.0027). A variant in the gene *MORC4* (rs12688220) showed significant interaction with alcohol (OR for homozygous and heterozygous risk allele -14.62 and 1.51 respectively, *P* = 0.0068) suggesting gene-environment interaction. A combined analysis of the genes *CLDN2* and *MORC4* based on an effective risk allele score revealed a higher percentage of individuals homozygous for the risk allele in CP cases with 5.09 fold enhanced risk in individuals with 7 or more effective risk alleles compared with individuals with 3 or less risk alleles (*P* = 1.88 x 10^-14^). Genetic variants in *CLDN2* and *MORC4* genes were associated with CP in Indian patients.

## Introduction

Chronic pancreatitis (CP) is a progressive and irreversible inflammatory disorder of pancreas leading to abdominal pain, diabetes and exocrine insufficiency. The prevalence of CP varies from 10 to 125/100000 individuals in different countries and is reported to be on much higher side in Indian population [1]. Various risk factors for CP are known such as alcohol, smoking, hereditary, metabolic but etiology is not known in the majority of patients termed as having Idiopathic chronic pancreatitis (ICP) [2, 3]. Even in those with known risk factors, the role is not fully explained by the injurious agent such as alcohol, as most other individuals with same risk factor do not develop CP. Thus, genetic susceptibility has been suggested to play an important role in etiopathogenesis of CP [4].

Indeed, starting with cationic trypsinogen gene (*PRSS1*) mutations in hereditary pancreatitis, mutations in many other genes predominantly *SPINK1*, *CFTR*, *chymotrypsin C*, *CPA1*, *CEL* etc. have been reported in patients with CP [5–9]. The risk susceptibility for CP cannot be fully explained by known genetic mutations. In this regard, a genome wide association study (GWAS) on pancreatitis identified polymorphisms at X-linked *CLDN2* locus being robustly associated with recurrent acute pancreatitis and predominantly alcohol-related chronic pancreatitis in North American patients of European ancestry [10].

Studies have reported various genetic variations in *SPINK1*, *CFTR*, *Cathepsin B* and *chymotrypsin C* genes being associated with CP in Indian patients [11–14]. However, these associations particularly of *SPINK1* gene are found in only up to 30–40% of patients and do not explain the disease risk in the majority of patients.

Various studies had revealed that genetic constitution of Indian population differs from other major ethnic population of the world [15]. Therefore, genes associated with a disorder in other populations need to be evaluated for their role in Indian population. In present study, we investigated recently identified GWAS variants associated with pancreatitis [10], in 1807 unrelated Indian individuals of Indo-European ethnicity for their association with CP.

## Methods

### Ethics Statement

Prior informed written consent was obtained from both patient and healthy control participants. The study was approved by the Human Ethics Committee of CSIR-Institute of Genomics and Integrative Biology, All India Institute of Medical Sciences research Ethics Committee; Ethics Committee of Post-graduate Institute of Medical Education and Research, Chandigarh; Sanjay Gandhi Post-graduate Institute of Medical Sciences research Ethics Committee, Lucknow and Institute of Post-graduate Medical Education and Research Ethics Committee, Kolkata. The study was conducted in accordance with the principles of Helsinki Declaration.

### Study design

This study involved 1807 unrelated Indian individuals of Indo-European ethnicity, including 519 patients with CP and 1288 ethnically matched healthy controls. Among the 519 CP patients, 434 subjects were with ICP and 85 were with Alcoholic chronic pancreatitis (ACP). The case subjects were recruited from the Departments of Gastroenterology at the following centers: All India Institute of Medical Sciences, New Delhi; Post-graduate Institute of Medical Education and Research, Chandigarh; Sanjay Gandhi Post-graduate Institute of Medical Sciences, Lucknow and Institute of Post-graduate Medical Education and Research, Kolkata by INDIPAN (Indian Consortium for Pancreatitis) members. The diagnosis and etiology of CP were made as described previously by Midha *et al*., 2009 [12]. Briefly, the diagnosis was made in the appropriate clinical setting if there was evidence of pancreatic duct dilatation and irregularity and/or pancreatic calcification on imaging studies that included ultrasonography, endoscopic retrograde cholangiopancreatography (ERCP); contrast enhanced computed tomography (CECT) scan, and/or magnetic resonance cholangiopancreatography (MRCP). The etiology of CP was determined as follows: (a) Alcoholic CP: If a patient was drinking >60 g (females) or >80 g (males) of alcohol per day for >5 years, (b) Hereditary CP: If >2 first-degree relatives were suffering from CP with an autosomal dominant pattern of inheritance; and those without autosomal dominant inheritance were labeled as familial chronic pancreatitis (c) Metabolic: If there was evidence of hyperparathyroidism or hypertriglyceridemia, (d) Traumatic: If there was a history of definite abdominal trauma with imaging evidence of pancreatic injury and subsequent ductal dilatation, and (e) Idiopathic: If no definite cause of CP was identified.

All control subjects of the current study are part of the INDICO (Indian Diabetes Consortium) [16]. The inclusion and exclusion criteria for control subjects were as described earlier [15], controls were ≥ 40 years aged healthy persons with no family history of diabetes and no visible symptoms of pancreatitis. They had fasting glucose level <110mg/dl and HbA1c level ≤ 6%. All patients underwent a detailed questionnaire based evaluation and extensive diagnostic work-up. In particular, data regarding age at onset of pancreatitis, age at diagnosis, duration of disease, family history of pancreatitis and known risk factors for chronic pancreatitis such as alcohol and smoking, and complications of CP such as diabetes were recorded.

### Genotyping

Out of the 11 SNPs (S1 Table) (p value<10^-7^ for the stage 1 or stage 2 or combined analysis) reported by Whitcomb *et al*. [10], 9 were taken for genotyping including two proxy SNPs. Two SNPs (rs7057398 and rs5917027) were not included in the study because these did not get multiplexed. Two proxies (rs12012022, proxy for rs12014762 and rs1985888/rs2855983, proxy for rs10273639) were used in this study because the original SNPs did not get multiplexed. Two SNPs (rs1985888 and rs2855983) were in LD with each other. Finally, we worked with 9 SNPs for all analysis. Genotyping of the cases was done using Sequenom MassARRAY^®^ system (iPLEX GOLD) (Sequenom, San Diego, CA, USA) following the manufacturer’s protocol and controls were genotyped on Illumina Human610-Quad Bead Chips (Illumina Inc., San Diego, CA). The quality control of genotype data has been as reported earlier [15]. Some of control samples (~3%) were genotyped using Sequenom MassARRAY^®^ to determine consistency in genotyping platforms and >95% genotype consistency was observed in both platforms. The genotype results of each plate were accepted only if the concordance rate of >99% was observed among duplicates. Quality control criteria for the SNPs to qualify for further analysis included: minor allele frequency (MAF) ≥0.05, missingness per SNP <5% and no significant deviation from the Hardy-Weinberg equilibrium (HWE).

### Statistical analysis

Association of the SNPs with CP was investigated for the whole group of CP patients and also separately for ICP and ACP. Genotype distribution for all SNPs was analyzed for deviation from HWE using χ^2^ analysis. Logistic regression analysis, assuming log additive model, to determine the association between SNPs and the risk for CP was performed. The associations were adjusted for sex and age as appropriate and OR with 95% CI are presented with respect to the allele as reported in initial study [10]. A *P* value of <0.0055 (α = 0.05/9) for 9 independent SNPs was considered significant for association with CP after Bonferroni correction for multiple testing. Association analysis was performed using following categories: ACP and ICP combined, ICP alone and ACP alone versus controls. For analysis of sex chromosome SNPs, male hemizygous genotypes were assumed as equivalent to female homozygous genotypes and coded as 0 and 2 for computational ease. As PLINK sets the count of minor alleles in males as 0 and 1, and includes a sex effect, using above coding has no impact on association tests based on logistic regression [17]. SNPs with OR <1 are considered protective while those with OR >1 are considered as conferring risk for the disease. The combined effect of the SNPs on the risk of CP was assessed by computing effective number of risk alleles, as described earlier [18]. Briefly, only SNPs with p<0.0055 (corrected *P* value) were used to calculate effective number of risk alleles. The weighted risk given by ΣwiXi/Σw_i_ (where X_i_ is the genotype coding for the i^th^ SNP as provided above and w_i_ is the logarithm of the odds Ratio corresponding to that SNP) was multiplied by 14 (maximum possible number of risk alleles corresponding to 7 SNPs found significant in present study) to obtain the ‘effective’ number of risk alleles. The combined risk analysis for *CLDN2* and *MORC4* genes was based on 4 SNPs and hence, effective number of risk alleles was obtained by multiplying weighted risk by 8. We note that while the maximum number of risk alleles at these 4 SNPs can only be 4 for males, the coding of 0 or 2 for hemizygous male genotypes allow for effective number of risk alleles to be computed as above. The analysis included only those individuals whose genotypes at all 7 SNPs (or 4 for *CLDN2* and *MORC4* locus) were available. Considering the individuals with <5 risk alleles as the reference (or <3 for *CLDN2* and *MORC4* locus), ORs and *P* values for increase in disease risk with each ‘effective’ risk allele were calculated after adjusting for age and sex. With adjustment for sex, the results of combined risk analysis would be identical to those obtained when effective number of risk alleles is computed based on the actual number of risk alleles at 4 SNPs (that is, by coding male hemizygous genotypes as 0 or 1). The genotype information of samples for studied SNPs has been given in S4 Table.

To further rule out the possibility of spurious association because of coding of male hemizygote as being male homozygote, sex matched case control analysis was performed for both ACP and ICP combined samples and ICP samples separately and meta-analysis of the summary statistics were done.

Gene-environment interaction of alcohol and smoking, with CP was estimated using generalized linear models (additive model using CGEN package in R). For 3 SNPs (rs11988997, rs12008279, rs6622126), above model did not fit and data for interaction with alcohol were analyzed under a recessive mode of inheritance and an additive risk model comprising the main effects of genotype, alcohol consumption and their interaction effect. Additionally, the interaction of SNP (rs11988997) with smoking was also analyzed using a similar additive risk model and χ^2^ test was used to determine significance of combined effect of SNPs at *CLDN2* and *MORC4* locus with CP. Statistical analyses were performed using R (version 3.0.1) and PLINK (http://pngu.mgh.harvard.edu/purcell/plink/).

## Results

### Study demographics

Of the 519 patients with CP, 434 had ICP and 85 had ACP as shown in Table 1. Patients with ICP developed disease at a significantly younger age (mean age at onset 23.42 years) compared with ACP patients (mean age 37.58 years; p<0.0001). Of the patients with ICP, 63.36% were males while all ACP patients were males, compared to 53.42% males in the controls (Table 1).

**Table 1:** Demographic data of patients with ICP, ACP and controls used in the study.

	Controls		Cases			
			ICP		ACP	
**Variable**	*n*		*n*		*n*	
***n* (men/women)**	1288	688/600	434	275/159	85	85/0
**Age (years)ⱡ**	1288	52.61 ± 10.37	432	30.12 ± 11.12*	78	42.78 ± 8.72*
**Age of onset (years)ⱡ**		-	410	23.42 ± 11.03	78	37.58 ± 8.72**
**Alcohol consumption (%)**	1286	192 (14.93)	410	22 (5.37)	85	85 (100)
**Smoking (%)**	1285	324 (25.21)	281	14 (4.98)	76	62 (81.58)
**Tobacco chewing (%)**	1285	168 (13.07)	281	7 (2.49)	76	8 (10.53)

n = Total number of samples with measured phenotype, ⱡdata are mean ± SD,

*p value <0.0001 showing significance vs. controls,

**p value<0.0001 showing significance vs. ICP cases.

#### Association analysis for CP (ACP and ICP combined) vs. controls

Comparing CP vs. controls, 4 genetic variations in X-linked *CLDN2* and *MORC4* genes showed significant association with CP and remained so even after adjustment for age and sex (Table 2). The gene variant for *MORC4* conferred highest susceptibility at rs12688220 (OR—1.72; 95% CI [1.43–2.06], P = 9.20 x 10^-09^) and at rs6622126 (OR—1.75, 95% CI [1.34–2.28], P = 4.04 x 10^-05^). Another variant at rs12012022 had an OR of 1.27 (95% CI [1.05–1.53]) but did not reach required statistical significance (P = 0.012). Two variants for *CLDN2* gene at rs4409525 (OR—1.71, 95% CI [1.44–2.04]; P = 1.38 x 10^-09^) and at rs12008279 (OR—1.56, 95% CI [1.24–1.96]; P = 1.53 x 10^-04^) also conferred significant risk. Furthermore, another variant at rs379742 for *MUM1L1-CXorf57* was also found to be significantly associated with CP (OR 1.32, 95% CI [1.11–1.58]; P = 0.0023), that is in consistence with earlier reports.

**Table 2:** Association of SNPs with CP (n = 519) and control (n = 1288) subjects.

CHR	Gene (SNP)	A1*	A2	Frequency		OR (95%CI)	SE	Punadj	OR (95%CI)	SE	Padj
				Cases	Controls						
7	*PRSS1-PRSS2* (rs2855983)	A	G	0.56	0.66	0.66 (0.57–0.76)	0.08	3.50 x 10^-08^	0.60 (0.48–0.76)	0.11	9.92 x 10^-06^
8	*SAMD12-TNFRSF11B* (rs11988997)	T	C	0.06	0.09	0.57 (0.42–0.78)	0.16	3.61 x 10^-04^	0.49 (0.31–0.78)	0.24	2.70 x 10^-03^
10	*KIAA1462-MTPAP* (rs2995271)	C	T	0.39	0.35	1.20 (1.03–1.40)	0.08	0.02	1.21 (0.96–1.52)	0.12	0.11
X	*MUM1L1-CXorf57* (rs379742)	T	C	0.45	0.32	1.47 (1.30–1.66)	0.06	1.03 x 10^-09^	1.32 (1.11–1.58)	0.09	2.30 x 10^-03^
X	*CLDN2* (rs4409525)	A	G	0.70	0.49	1.84 (1.62–2.08)	0.06	5.71 x 10^-22^	1.71 (1.44–2.04)	0.09	1.38 x 10^-09^
X	*CLDN2* (rs12008279)	G	A	0.89	0.73	2.05 (1.71–2.46)	0.09	7.27 x 10^-15^	1.56 (1.24–1.96)	0.12	1.53 x 10^-04^
X	*MORC4* (rs12012022)	T	C	0.36	0.25	1.44 (1.27–1.64)	0.07	1.87 x 10^-08^	1.27 (1.05–1.53)	0.09	0.01
X	*MORC4* (rs6622126)	A	G	0.89	0.72	2.42 (1.95–3.01)	0.11	1.16 x 10^-15^	1.75 (1.34–2.28)	0.14	4.04 x 10^-05^
X	*MORC4* (rs12688220)	T	C	0.73	0.48	1.96 (1.71–2.24)	0.07	3.41 x 10^-22^	1.72 (1.43–2.06)	0.09	9.20 x 10^-09^

* Effect sizes of the present study are presented with respect to the reported allele (A1) in the source study, for proxy SNPs the allele on same strand as that of reported allele has been used. Significance achieved at P = 0.05/9 (α = 0.0055) after Bonferroni correction.

CHR = chromosome, SNP = single nucleotide polymorphism, A2 = Other allele, OR = odds ratio, CI = confidence interval, SE = Standard error, Punadj = unadjusted analysis and Padj = adjusted analysis for covariates. Analysis is adjusted for sex and age.

We also found significant association at *PRSS1-PRSS2* locus with OR 0.60 (95% CI [0.48–0.76], P = 9.92 x 10^-06^) as protective for CP. In addition, another variant at rs11988997 for *SAMD12-TNFRSF11B* was also found to be protective (OR 0.49, 95% CI [0.31–0.78]; P = 0.0027). Sex-stratified analysis also showed similar results as shown in S2 Table.

#### Association analysis for ICP vs. controls

In patients with ICP, all 5 variants at X-linked *CLDN2* and *MORC4* genes retained significant association with susceptibility to ICP with ORs ranging from 1.41 to 2.42 and *P* values ranging from 9.85 x 10^-07^ to 1.24 x 10^-19^ (Table 3). Following adjustment for age and sex, variants at rs6622126 (OR 1.62, 95% CI [1.16–2.25]; P = 0.0042), rs4409525 (OR 1.57, 95% CI [1.28–1.93]; P = 1.36 x 10^-05^) and rs12688220 (OR 1.50, 95% CI [1.20–1.87]; P = 2.98 x 10^-04^) had significant association with ICP.

**Table 3:** Association of SNPs with CP in ICP cases (n = 434) and control (n = 1288) subjects.

CHR	Gene (SNP)	A1*	A2	Frequency		OR (95%CI)	SE	Punadj	OR (95%CI)	SE	Padj
				Cases	Controls						
7	*PRSS1-PRSS2* (rs2855983)	A	G	0.58	0.66	0.69 (0.59–0.81)	0.08	4.57 x 10^-06^	0.63 (0.49–0.82)	0.13	5.78 x 10^-04^
8	*SAMD12-TNFRSF11B* (rs11988997)	T	C	0.06	0.09	0.60 (0.44–0.84)	0.17	2.20 x 10^-03^	0.54 (0.31–0.92)	0.28	0.02
10	*KIAA1462-MTPAP* (rs2995271)	C	T	0.38	0.35	1.15 (0.98–1.36)	0.08	0.09	1.08 (0.83–1.42)	0.14	0.56
X	*MUM1L1-CXorf57* (rs379742)	T	C	0.46	0.32	1.52 (1.33–1.73)	0.07	4.91 x 10^-10^	1.31 (1.06–1.62)	0.11	0.01
X	*CLDN2* (rs4409525)	A	G	0.70	0.47	1.86 (1.62–2.12)	0.07	1.24 x 10^-19^	1.57 (1.28–1.93)	0.10	1.36 x 10^-05^
X	*CLDN2* (rs12008279)	G	A	0.89	0.73	2.04 (1.68–2.48)	0.10	7.68 x 10^-13^	1.46 (1.12–1.91)	0.14	5.80 x 10^-03^
X	*MORC4* (rs12012022)	T	C	0.36	0.25	1.41 (1.23–1.61)	0.07	9.85 x 10^-07^	1.14 (0.92–1.43)	0.11	0.23
X	*MORC4* (rs6622126)	A	G	0.89	0.72	2.42 (1.91–3.06)	0.12	2.04 x 10^-13^	1.62 (1.16–2.25)	0.17	4.20 x 10^-03^
X	*MORC4* (rs12688220)	T	C	0.73	0.48	1.91 (1.65–2.21)	0.07	7.02 x 10^-18^	1.50 (1.20–1.87)	0.11	2.98 x 10^-04^

* Effect sizes of current study are presented with respect to the reported allele (A1) in the source study, for proxy SNPs the allele on same strand as that of reported allele has been used. Significance achieved at P = 0.05/9 (α = 0.0055) after Bonferroni correction.

CHR = chromosome, SNP = single nucleotide polymorphism, A2 = Other allele, OR = odds ratio, CI = confidence interval, SE = Standard error, Punadj = unadjusted analysis and Padj = adjusted analysis for covariates. Analysis is adjusted for sex and age.

We also found that the variant rs2855983 at *PRSS1-PRSS2* locus was significantly associated in ICP patients similar to its status in the combined analysis (OR 0.63, 95% CI [0.49–0.82]; P = 5.78 x 10^-04^). Sex-stratified analysis also showed similar results as shown in S3 Table.

#### Association analysis for ACP vs. controls

In the ACP patients, all 5 variants in X-linked *CLDN2* and *MORC4* genes showed significant association with ACP even after adjustment for age and sex (Table 4). Similar to combined analysis, *CLDN2* and *MORC4* gene locus showed conferment of susceptibility in ACP group with ORs ranging from 1.48 to 2.00 and *P* value 0.003 to 1.89 x 10^-06^ (Table 4).

**Table 4:** Association of SNPs with CP in ACP cases (n = 85) and control (n = 1288) subjects.

CHR	Gene (SNP)	A1*	A2	Frequency		OR (95%CI)	SE	Punadj	OR (95%CI)	SE	Padj
				Cases	Controls						
7	*PRSS1-PRSS2* (rs2855983)	A	G	0.51	0.66	0.53 (0.39–0.72)	0.16	5.94 x 10^-05^	0.55 (0.39–0.79)	0.18	1.20 x 10^-03^
8	*SAMD12-TNFRSF11B* (rs11988997)	T	C	0.04	0.09	0.43 (0.20–0.92)	0.39	0.03	0.46 (0.20–1.04)	0.42	0.06
10	*KIAA1462-MTPAP* (rs2995271)	C	T	0.44	0.35	1.46 (1.05–2.03)	0.17	0.02	1.54 (1.06–2.25)	0.19	0.02
X	*MUM1L1-CXorf57* (rs379742)	T	C	0.40	0.32	1.25 (0.95–1.65)	0.14	0.11	1.30 (0.99–1.70)	0.14	0.06
X	*CLDN2* (rs4409525)	A	G	0.69	0.47	1.84 (1.41–2.42)	0.14	9.75 x 10^-06^	1.82 (1.39–2.39)	0.14	1.14 x 10^-05^
X	*CLDN2* (rs12008279)	G	A	0.90	0.73	2.23 (1.44–3.46)	0.22	3.46 x 10^-04^	1.76 (1.19–2.60)	0.20	4.60 x 10^-03^
X	*MORC4* (rs12012022)	T	C	0.40	0.25	1.64 (1.26–2.13)	0.13	2.00 x 10^-04^	1.48 (1.14–1.93)	0.13	3.40 x 10^-03^
X	*MORC4* (rs6622126)	A	G	0.91	0.72	2.53 (1.52–4.20)	0.26	3.36 x 10^-04^	1.94 (1.25–3.00)	0.22	3.00 x 10^-03^
X	*MORC4* (rs12688220)	T	C	0.77	0.48	2.28 (1.69–3.08)	0.15	7.49 x 10^-08^	2.00 (1.50–2.66)	0.15	1.89 x 10^-06^

*Effect sizes of current study are presented with respect to the reported allele (A1) in the source study, for proxy SNPs the allele on same strand as that of reported allele has been used. Significance achieved at P = 0.05/9 (α = 0.0055) after Bonferroni correction.

CHR = chromosome, SNP = single nucleotide polymorphism, A2 = Other allele, OR = odds ratio, CI = confidence interval, SE = Standard error, Punadj = unadjusted analysis and Padj = adjusted analysis for covariates. Analysis is adjusted for age.

Similar to the ICP patients, a significant association at *PRSS1-PRSS2* locus with OR 0.55 (95% CI [0.39–0.79], P = 0.001) was present in ACP patients.

### Combined risk analysis

**Fig 1:**
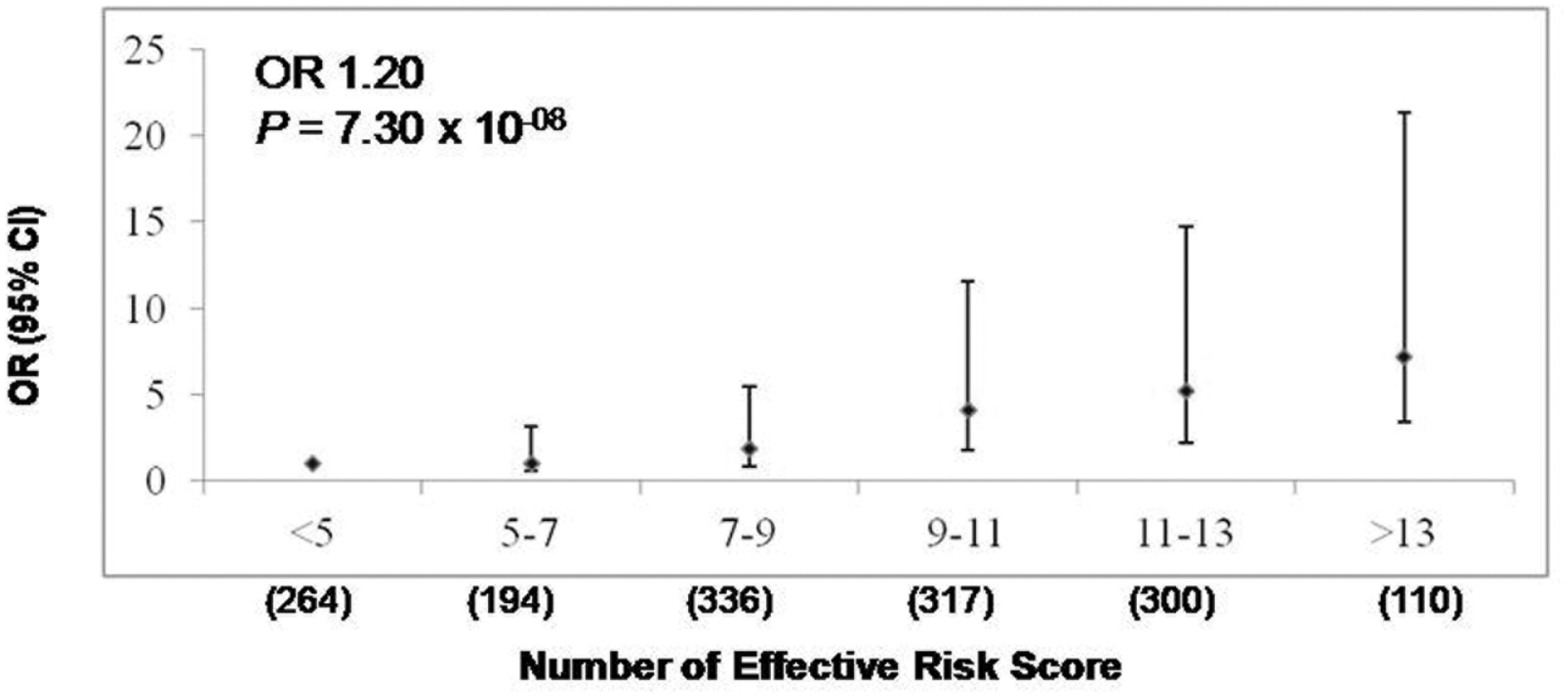
Combined risk analysis to determine the effect of increase in number of ‘effective’ risk alleles with risk of CP. On *y*-axis OR and 95% CI are plotted, corresponding to number of effective risk alleles on the *x*-axis. Number in parenthesis on the *x*-axis indicates Sample size in each category. For every unit increase in the number of effective’ risk allele, the OR is 1.20 and *P* value 7.30 x 10^-08^.

The analysis based on the effective number of risk alleles of all 7 significant SNPs (at p < 0.0055), revealed significantly enhanced risk of CP by 1.2 fold with increase in each unit of ‘effective’ risk allele (*P* = 7.3 x 10^-08^) (Fig 1). Individuals with 13 or more risk alleles carried 7.17 fold increased risk for CP compared with the individuals with 5 or less risk alleles (*P* = 7.51 x 10^-12^).

### Assessment of variation at *CLDN2* and *MORC4* locus

**Fig 2:**
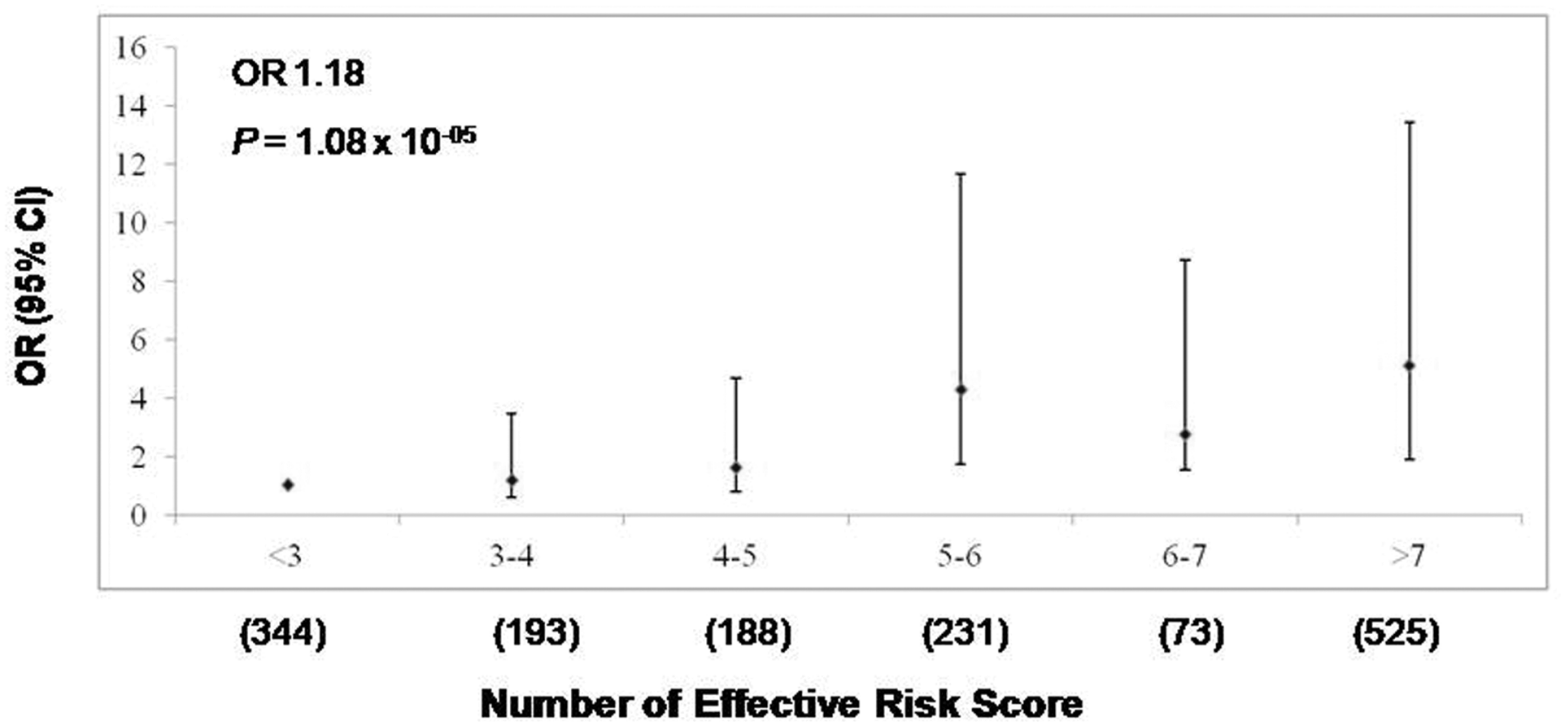
Combined risk analysis to determine the effect of increase in number of ‘effective’ risk alleles at *CLDN2* and *MORC4* loci with risk of CP. On *y*-axis OR and 95% CI are plotted, corresponding to number of effective risk alleles on the *x*-axis. Number in parenthesis on the *x*-axis indicates Sample size in each category. For every unit increase in the number of effective’ risk allele, the OR is 1.18 and *P* value 1.08 x 10^-05^.

As, *CLDN2*, MORC4 genes are positioned closely and showed strongest association with risk of CP, a detailed analysis of SNPs within the *CLDN2* and *MORC4* genes, termed *CLDN2* and *MORC4* locus was performed. Homozygous individuals for risk alleles were more prevalent in cases versus controls (Table 5). Having either one or more variation within the locus increased the risk of CP substantially by 3.40 fold (95% CI [2.00–6.17], P = 1.37 x 10^-06^) in cases. The combined risk analysis of 4 significantly associated SNPs (at p < 0.0055), showed an increase in risk of CP by 1.18 fold for increase in each unit of ‘effective’ risk allele (*P* = 1.08 x 10^-05^) (Fig 2) with 5.09 fold enhanced risk in females with 7 (3.5 in males) or more effective number of risk alleles compared with females with 3 (1.5 in males) or less effective number of risk alleles (*P* = 1.88 x 10^-14^).

**Table 5:** Comparison of SNPs at *CLDN2* and *MORC4* locus in CP cases and controls.

		Cases					Control				
		*n*			%		*n*			%	
Gene (SNP)	Risk allele	Total	Hom	Het	Hom	Het	Total	Hom	Het	Hom	Het
*CLDN2* (rs4409525)	A	503	327	45	65.01	8.95	1284	446	295	34.74	22.98
*CLDN2* (rs12008279)	G	502	434	29	86.45	5.78	1282	829	230	64.66	17.94
*MORC4* (rs12012022)	T	498	128	109	25.70	21.89	1286	200	241	15.55	18.74
*MORC4* (rs6622126)	A	380	327	33	86.05	8.68	1286	801	240	62.29	18.66
*MORC4* (rs12688220)	T	441	283	70	64.17	15.87	1279	462	300	36.12	23.46

Hom = Homozygote for risk allele, Het = Heterozygote, n = Number of samples

### Gene-Environment interaction analysis

Interaction of genetic variants with environmental factors showed significant effect of rs12688220 (*MORC4*) with alcohol (OR 14.62 for interaction of homozygous and OR 1.51 for interaction of heterozygous risk allele, *P* = 0.0068) hence amplifying the risk of CP (Table 6). No significant interaction of any other risk alleles was observed with either alcohol or smoking in CP patients.

**Table 6:** Gene-Environment interaction of genetic variants with alcohol and smoking in CP.

**Alcohol**							
		**OR**		**Punadj**	**OR**		**Padj**
**Gene (SNP)**	**Risk/ non-risk allele**	**Homozygous**	**Heterozygous**		**Homozygous**	**Heterozygous**	
*PRSS1-PRSS2* (rs2855983)	G/A	1.59	1.89	0.18	1.41	2.81	0.05
*SAMD12-TNFRSF11B* (rs11988997)	C/T	1.17	-	0.54	1.54	-	0.19
*MUM1L1-CXorf57* (rs379742)	T/C	2.74	0.51	0.26	3.48	0.93	0.57
*CLDN2* (rs4409525)	A/G	2.56	0.80	0.61	8.94	2.14	9.80 x 10^-03^
*CLDN2* (rs12008279)	G/A	0.95	-	0.55	1.19	-	0.21
*MORC4* (rs6622126)	A/G	0.82	-	0.64	1.28	-	0.10
*MORC4* (rs12688220)	T/C	2.63	0.67	0.36	14.62	1.51	6.80 x 10^-03^
**Smoking**							
		**OR**		**Punadj**	**OR**		**Padj**
**Gene (SNP)**	**Risk/ non-risk allele**	**Homozygous**	**Heterozygous**		**Homozygous**	**Heterozygous**	
*PRSS1-PRSS2* (rs2855983)	G/A	2.74	2.15	0.20	1.78	1.87	0.26
*SAMD12-TNFRSF11B* (rs11988997)	C/T	1.30	-	0.71	1.59	-	0.32
*MUM1L1-CXorf57* (rs379742)	T/C	1.51 x 10^-06^	0.37	3.90 x 10^-03^	2.26 x 10^-06^	0.38	0.27
*CLDN2* (rs4409525)	A/G	3.32 x 10^-06^	1.08	0.49	4.75 x 10^-06^	1.47	0.27
*CLDN2* (rs12008279)	G/A	2.27 x 10^-06^	0.65	0.29	3.37 x 10^-06^	0.85	0.70
*MORC4* (rs6622126)	A/G	1.53 x 10^-06^	0.42	0.11	2.06 x 10^-06^	0.61	0.66
*MORC4* (rs12688220)	T/C	0.61	0.69	0.28	2.59	0.91	0.63

Risk allele is used for calculation of odds ratio and P value. Significance achieved at P = 0.05/7 (α < 0.0071) for the 7 significantly associated variants. SNP = single nucleotide polymorphism, OR = odds ratio, Punadj = unadjusted analysis and Padj = adjusted analysis for covariates.

## Discussion

In present study, we examined the association of 9 SNPs, identified in a recent GWAS on pancreatitis [10], in 1807 Indian subjects. Whitcomb *et al*., reported *CLDN2* and *PRSS1-PRSS2* loci to be associated with recurrent acute pancreatitis and CP (predominantly alcohol-related chronic pancreatitis) in North American subjects of European ancestry. This work confirms association of variants in X-linked *CLDN2* and *MORC4* gene with risk susceptibility in Indian patients with CP. The association might be more robust for CP since we included well characterized patients with CP only and not those with recurrent acute pancreatitis.

GWAS by Whitcomb et al., recently reported association of *CLDN2* gene variants with CP in Chinese, European, Japanese and Indian patients [19–22]. Interestingly, our study confirms the same in Indian CP patients of Indo-European ethnicity. *CLDN2* gene codes for protein claudin-2 that lies in tight junctions and immunohistochemical staining showed weak but membrane-bound expression in acinar, ductal and islet cells in normal human pancreatic tissue [23]. However, premalignant pancreatic cystic lesions showed a more robust expression of claudin-2 suggesting that its aberrant form may directly interfere with tight junction formation and function. Whether the variants in the gene might be related to impaired permeability, affecting either acinar or ductal cells function remains to be explored. Additionally, *CLDN2* promoter includes a nuclear factor κB (NF-κB) binding site [24] and a variant *CLDN2* might have a role in NF-kB activation.

*MORC4* shows wide spread expression in normal tissues including a weak but positive signal in human pancreas [25] and Liggins *et al*., identified its protein to contain a highly conserved N-terminus, an ATPase domain that may indicate a functional requirement for ATP hydrolysis, a zinc finger motif that may promote protein–protein or protein–nucleic acid interactions and a CW zinc finger with a probable role with methylated DNA or chromatin [25]. A Swedish study reported significant association between risk to Crohn's disease and SNP in *MORC4* (rs6622126, also significantly associated with CP in present study) using a case-control approach [26]. Our findings suggest an increased risk of CP with variants in *MORC4* gene in addition to a high risk coupled with alcohol intake suggesting a strong gene environment interaction. Polymorphisms of alcohol metabolizing genes have not explained the susceptibility of some individuals to develop pancreatitis. If proven, such a gene environment interaction could explain individual susceptibility and possibly organ specificity. The downstream signaling of *MORC4* activation in pancreatitis needs exploration using experimental models but a protein-protein interaction database (Protein Interaction Network Analysis; PINA) [27] suggests its interaction with AMPKa1, GEMIN4, HECW2, SKIL, STAT3 and UBC, and downstream regulation of all/either of these might be pathophysiologically relevant.

It seems likely that *CLDN2* and/or *MORC4* genes play an important pathophysiological role in CP. Association of X-linked genetic variants with CP might be an important reason for male predominance among the patients with CP as has also been suggested by Whitcomb *et al* [10].

Although gain-of-function mutations in gene for cationic trypsinogen (*PRSS1*) are well established in hereditary pancreatitis in patients of different ethnicity [28,29], previous studies in Indian patients with CP did not identify any significant mutations/polymorphisms in the *PRSS1* gene [11,12,30,31]. Interestingly, a loss-of-function mutation (p.G191R) in anionic trypsinogen gene (*PRSS2*) has been reported protective against developing pancreatitis in European, Japan and Indian populations [32–35]. A current investigation identified a functional promoter variant rs4726576C/A near rs10273639 to affect the expression of *PRSS1* gene in pancreatic tissue in mice [36]. Significant association of protective variant in *PRSS1*-*PRSS2* locus in all three categorical analyses i.e. ICP and ACP combined as well as only ICP/ ACP versus controls suggesting a role of *PRSS1*-*PRSS2* locus in Indian patients.

We also found significant association of variant (rs11988997) in *SMAD12-TNFRSF11B* conferring a protective status to CP in our population. *SAMD12* (Sterile Alpha Motif Domain containing 12) proteins containing SAM domains show varied protein–protein interaction nodes, though a conclusive function has not been assigned to this gene [37]. *TNFRSF11B* (gene for osteoprotegerin, OPG) is a member of TNF-receptor super family and its expression was shown to correlate with the aggressiveness of pancreatic ductal cell carcinoma [38]. Using a rat beta cell line and human primary pancreatic islets, OPG was shown to prevent cytokine induced p38 MAPK phosphorylation and subsequent beta cell death, and suggested to act as an autocrine or paracrine survival factor for beta cells [39]. On the contrary, a recent study demonstrated the role of serum OPG in promoting beta cell dysfunction *in vivo* in mice [40] and therefore its role in pancreatic structure and function needs further investigation. There are no other reports showing association of this gene with CP.

## Conclusion

We replicated association of common variants in *CLDN2*, *MORC4*, *PRSS1-PRSS2* and *SMAD12-TNFRSF11B* loci with CP in Indians subjects of Indo-European origin and showed significant association particularly with variants in the *MORC4/CLDN2* locus. Further studies through deep sequencing are required to identify the actual polymorphism/mutation associated with CP as are functional studies to elucidate the mechanism of injury.

## Supporting Information

S1 Table List of SNPs selected in the present study from source study. The R^2^ value has been given on the basis of Human Genome version 17.
(DOCX)

S2 Table Association analysis and meta-analysis result of (ACP+ICP) verses control samples stratified by sex. Meta-analysis was done using PLINK for summary statistics of male and female. *Effect sizes of current study are presented with respect to the reported allele (A1) in the source study, for proxy SNPs the allele on same strand as that of reported allele has been used. Significance achieved at P = 0.05/9 (α = 0.0055) after Bonferroni correction. SNP = single nucleotide polymorphism, OR^f^ = odds ratio for fixed-effects meta-analysis, P^f^ = P value for fixed effect meta-analysis, CI = confidence interval, Q = P value for Cochrane Q statistic.
(DOCX)

S3 Table Association analysis and meta-analysis result of ICP verses controls samples stratified by sex. Meta-analysis was done using PLINK for summary statistics of male and female. *Effect sizes of current study are presented with respect to the reported allele (A1) in the source study, for proxy SNPs the allele on same strand as that of reported allele has been used. Significance achieved at P = 0.05/9 (α = 0.0055) after Bonferroni correction.
SNP = single nucleotide polymorphism, OR^f^ = odds ratio for fixed-effects meta-analysis, P^f^ = P value for fixed effect meta-analysis, CI = confidence interval, Q = P value for Cochrane Q statistic.
(DOCX)

S4 Table Raw genotype information for samples for all studied SNPs. *Sex chromosomal SNP. For sex chromosomal SNP, genotype for male hemizygote has been coded as homozygote genotype for corresponding allele. Sex (1 = Male; 2 = Female), Pheno (1 = Case, 2 = Control). http://dx.doi.org/10.5061/dryad.4t9f3
(DOCX)

## Abbreviations

CP: Chronic Pancreatitis
GWAS: Genome-Wide Association Study
ICP: Idiopathic Chronic Pancreatitis
ACP: Alcoholic Chronic Pancreatitis
LD: Linkage Disequilibrium
MAF: Minor Allele Frequency
HWE: Hardy-Weinberg Equilibrium
OR: Odds Ratio
SNP: Single Nucleotide Polymorphism
CI: Confidence Interval
