# Supplementary material for: Common Variants in CLDN2 and MORC4 Genes Confer Disease Susceptibility in Patients with Chronic Pancreatitis
Source: PLoS One. 2016 Jan 28;11(1):e0147345. doi: 10.1371/journal.pone.0147345 (PMC4731142; doi:10.1371/journal.pone.0147345)
Supplement: S1 Table — The R2 value has been given on the basis of Human Genome version 17. (DOCX) [file pone.0147345.s001.docx]

**S1 Table:** **List of SNPs selected in the present study from source study.**

| **CHR** | **Gene** | **SNP** |  | **Proxy** | **R^2^** | **Location** |
| --- | --- | --- | --- | --- | --- | --- |
| 7 | *PRSS1-PRSS2* | rs10273639 | not multiplexed | rs2855983* | 0.90 | Intron |
|  | *PRSS1* |  |  | rs1985888 | 0.93 |  |
| 8 | *SAMD12-TNFRSF11B* | rs11988997* |  |  |  | Intergenic |
| 10 | *KIAA1462-MTPAP* | rs2995271* |  |  |  | Intergenic |
| X | *MUM1L1-CXorf57* | rs379742* |  |  |  | Intergenic |
| X | *CLDN2* | rs4409525* |  |  |  | Intron |
| X | *CLDN2* | rs12008279* |  |  |  | Intron |
| X | *MORC4* (*CLDN2* locus) | rs12014762 | not multiplexed | rs12012022* | 1 | Intron |
| X | *MORC4* (*CLDN2* locus) | rs6622126* |  |  |  | Exon |
| X | *MORC4* (*CLDN2* locus) | rs12688220* |  |  |  | 5’ UTR |
| X | *RIPPLY1* (*CLDN2* locus) | rs7057398 | not multiplexed |  |  |  |
| X | *CLDN2* | rs5917027 | not multiplexed |  |  |  |
| *Used for analysis | |  |  |  |  |  |

The R^2^ value has been given on the basis of Human Genome version 17
