# Supplementary material for: Common Variants in CLDN2 and MORC4 Genes Confer Disease Susceptibility in Patients with Chronic Pancreatitis
Source: PLoS One. 2016 Jan 28;11(1):e0147345. doi: 10.1371/journal.pone.0147345 (PMC4731142; doi:10.1371/journal.pone.0147345)
Supplement: S2 Table — Meta-analysis was done using PLINK for summary statistics of male and female. *Effect sizes of current study are presented with respect to the reported allele (A1) in the source study, for proxy SNPs the allele on same strand as that of reported allele has been used. Significance achieved at P = 0.05/9 (α = 0.0055) after Bonferroni correction. SNP = single nucleotide polymorphism, ORf = odds ratio for fixed-effects meta-analysis, Pf = P value for fixed effect meta-analysis, CI = confidence interval, Q = P value for Cochrane Q statistic. (DOCX) [file pone.0147345.s002.docx]

**S2 Table: Association analysis and meta-analysis result of (ACP+ICP) verses control samples stratified by sex.**

|  |  |  | **Frequency** | | **Male** | | **Female** | | **Meta-analysis** | | |
| --- | --- | --- | --- | --- | --- | --- | --- | --- | --- | --- | --- |
| **SNP** | **A1*** | **A2** | **Males** | **Females** | **OR (95%CI)** | **P** | **OR (95%CI)** | **P** | **OR^f^** | **P^f^** | **Q** |
| rs2855983 | A | G | 0.62 | 0.66 | 0.60 (0.46 -0.78) | 1.5 x 10^-04^ | 0.60 (0.36 -0.78) | 0.01 | 0.59 | 5.2 x 10^-06^ | 0.81 |
| rs11988997 | T | C | 0.08 | 0.09 | 0.48 (0.28 -0.84) | 9.6 x 10^-03^ | 0.53 (0.22 -1.27) | 0.16 | 0.50 | 3.1 x 10^-06^ | 0.88 |
| rs2995271 | C | T | 0.37 | 0.35 | 1.30 (1.00 -1.71) | 0.06 | 1.00 (0.63 -1.59) | 0.99 | 1.21 | 0.09 | 0.34 |
| rs379742 | T | C | 0.35 | 0.35 | 1.30 (1.07 -1.58) | 9.6 x 10^-03^ | 1.41 (0.94 -2.13) | 0.10 | 1.32 | 2.4 x 10^-03^ | 0.72 |
| rs4409525 | A | G | 0.52 | 0.53 | 1.61 (1.33 -1.96) | 5.9 x 10^-07^ | 2.04 (1.33 -3.13) | 9.6 x 10^-04^ | 1.67 | 3.6 x 10^-09^ | 0.32 |
| rs12008279 | G | A | 0.80 | 0.76 | 1.59 (1.24 -2.04) | 3.8 x 10^-04^ | 1.37 (0.83 -2.27) | 0.22 | 1.54 | 2.0 x 10^-04^ | 0.62 |
| rs12012022 | T | C | 0.28 | 0.28 | 1.26 (1.03 -1.55) | 0.03 | 1.29 (0.85 -1.97) | 0.23 | 1.27 | 0.01 | 0.92 |
| rs6622126 | A | G | 0.87 | 0.74 | 1.82 (1.35 -2.50) | 8.9 x 10^-05^ | 1.39 (0.78 -2.50) | 0.27 | 1.75 | 6.7x 10^-06^ | 0.41 |
| rs12688220 | T | C | 0.54 | 0.54 | 1.64 (1.35 - 2.00) | 9.7 x 10^-07^ | 1.89 (1.19 -2.94) | 6.5x 10^-03^ | 1.67 | 2.4x 10^-08^ | 0.60 |

Meta-analysis was done using PLINK for summary statistics of male and female. *Effect sizes of current study are presented with respect to the reported allele (A1) in the source study, for proxy SNPs the allele on same strand has been used. Significance achieved at P =0.05/9 (α = 0.0055) after Bonferroni correction.

SNP = single nucleotide polymorphism, OR^f^ = odds ratio for fixed-effects meta-analysis, P^f^ = P value for fixed effect meta-analysis, CI=confidence interval, Q = P value for Cochrane Q statistic.
