# Supplementary material for: Common Variants in CLDN2 and MORC4 Genes Confer Disease Susceptibility in Patients with Chronic Pancreatitis
Source: PLoS One. 2016 Jan 28;11(1):e0147345. doi: 10.1371/journal.pone.0147345 (PMC4731142; doi:10.1371/journal.pone.0147345)
Supplement: S4 Table — *Sex chromosomal SNP. For sex chromosomal SNP, genotype for male hemizygote has been coded as homozygote genotype for corresponding allele. Sex (1 = Male; 2 = Female), Pheno (1 = Case, 2 = Control). http://dx.doi.org/10.5061/dryad.4t9f3 (DOCX) [file pone.0147345.s004.docx]

**S4 Table: Raw genotype information for samples for all studied SNPs.**

*Sex chromosomal SNP

For sex chromosomal SNP, genotype for male hemizygote has been coded as homozygote genotype for corresponding allele.

Sex (1=Male; 2=Female), Pheno (1=Case, 2=Control).

**http://dx.doi.org/10.5061/dryad.4t9f3**
